# Skull Defects in Finite Element Head Models for Source Reconstruction from Magnetoencephalography Signals

**DOI:** 10.3389/fnins.2016.00141

**Published:** 2016-04-07

**Authors:** Stephan Lau, Daniel Güllmar, Lars Flemming, David B. Grayden, Mark J. Cook, Carsten H. Wolters, Jens Haueisen

**Affiliations:** ^1^Institute of Biomedical Engineering and Informatics, Technical University IlmenauIlmenau, Germany; ^2^Department of Neurology, Biomagnetic Center, University Hospital JenaJena, Germany; ^3^NeuroEngineering Laboratory, Department of Electrical and Electronic Engineering, University of MelbourneParkville, VIC, Australia; ^4^Centre for Neural Engineering, University of MelbourneParkville, VIC, Australia; ^5^Department of Medicine – St. Vincent's Hospital, University of MelbourneFitzroy, VIC, Australia; ^6^Medical Physics Group, Department of Diagnostic and Interventional Radiology, University Hospital JenaJena, Germany; ^7^Institute for Biomagnetism and Biosignalanalysis, Westfälische Wilhelms-Universität MünsterMünster, Germany

**Keywords:** magnetoencephalography, skull, finite element analysis, electric conductivity, source analysis, validation study

## Abstract

Magnetoencephalography (MEG) signals are influenced by skull defects. However, there is a lack of evidence of this influence during source reconstruction. Our objectives are to characterize errors in source reconstruction from MEG signals due to ignoring skull defects and to assess the ability of an exact finite element head model to eliminate such errors. A detailed finite element model of the head of a rabbit used in a physical experiment was constructed from magnetic resonance and co-registered computer tomography imaging that differentiated nine tissue types. Sources of the MEG measurements above intact skull and above skull defects respectively were reconstructed using a finite element model with the intact skull and one incorporating the skull defects. The forward simulation of the MEG signals reproduced the experimentally observed characteristic magnitude and topography changes due to skull defects. Sources reconstructed from measured MEG signals above intact skull matched the known physical locations and orientations. Ignoring skull defects in the head model during reconstruction displaced sources under a skull defect away from that defect. Sources next to a defect were reoriented. When skull defects, with their physical conductivity, were incorporated in the head model, the location and orientation errors were mostly eliminated. The conductivity of the skull defect material non-uniformly modulated the influence on MEG signals. We propose concrete guidelines for taking into account conducting skull defects during MEG coil placement and modeling. Exact finite element head models can improve localization of brain function, specifically after surgery.

## Introduction

Localization of neuronal activity in the brain of patients is a common task in clinical neurophysiology. Specifically, during pre-surgical planning it is essential to locate physiological and pathophysiological brain activity as accurately as possible. Magnetoencephalography (MEG) is a non-invasive, functional recording modality that captures localizing information with high temporal resolution. Source reconstruction from MEG signals is a localization approach that reconstructs the distribution of neuronal currents inside the brain using a detailed volume conductor model of the patient's head.

Skull defects, such as post-surgical skull openings, are a challenge for functional evaluation of the brain. Initially, it was hypothesized that skull defects have a negligible influence on MEG signals and source reconstruction based on a small number of post-mortem phantom experiments, *in vivo* animal experiments, and simulation studies. Barth et al. (1986) used a physical coaxial dipole to simulate intracerebral currents in a formalin fixed human cranial specimen which was filled with conducting jelly. Okada et al. (1999b) recorded the MEG of a somatic evoked response in anesthetized piglets first over intact skull and then over the dura after a large skull section was removed (skull on vs. skull off). The main limitation of these experiments was that the skull defect was filled with non-conducting air instead of a conducting material mimicking the soft tissue in a healed skull defect. An early simulation of MEG above a 3 cm wide skull defect in a multi-sphere head model by Van den Broek et al. (1998) indicated that the MEG signals generated by sources placed next to a skull defect are not affected by the skull defect, and no source reconstruction errors could be observed for these source locations. However, sources under a skull defect remained an open question. Therefore, comprehensive evidence under realistic conditions that would allow one to generalize to post-surgical skull defects has been missing.

Recently, we reported on our *in vivo* animal experiment of the influence of conducting skull defects on MEG signals above and around two skull defects, using a well-defined current source under the middle and edge of one defect and next to it (Lau et al., 2014). The results demonstrated that skull defects can, in fact, substantially influence MEG signals and that the MEG signal magnitude deviates most if the source is under the middle of a skull defect. The change in MEG signal topography is dependent on the skull defect geometry and the relative position and orientation of the source. These measurements closed the gap in the experimental evidence. The question arises of whether volume conductor models of the head can reproduce these MEG signal changes accurately and whether source reconstruction from MEG in the presence of skull defects is possible.

The finite element (FE) method allows us to discretize the head volume into small volume elements and, therefore, to differentiate many tissue types and skull defects. Simulations by Vorwerk et al. (2014) showed that incorporating fine anatomical detail of a head with intact skull influences MEG signals and source reconstruction. Using this detailed modeling approach, Lew et al. (2013) investigated MEG in a FE model of a neonate head with and without differentiation of fontanels and sutures, which are natural skull defects. They reported relatively small differences in the MEG signals and source reconstruction for realistic source patches due to ignoring fontanels and sutures in the head model. The neonate skull is different from the adult one, because it is much thinner and of much higher conductivity. Consequently, the influence of non-neonate skull defects on MEG signals and source reconstruction remains to be modeled and evaluated. The MEG measurements of our controlled experiment (Lau et al., 2014) present a unique opportunity to validate a detailed FE model of skull defects with respect to corresponding *in vivo* MEG measurements and known source positions (Sander et al., 2010).

Therefore, the objectives of this study are (1) to assess the concordance of a detailed FE simulation of the controlled MEG skull defect experiment with the physical measurements of Lau et al. (2014), (2) to describe errors in source reconstruction from measured MEG signals caused by ignoring skull defects, (3) to assess the ability of an exact FE head model to overcome such source reconstruction errors, and (4) to identify critical modeling steps for skull defects.

## Materials and methods

### Experimental data

With ethics approval (Freistaat Thüringen, Germany, 02 034/08) we performed a 16-channel MEG of signals produced by a miniaturized artificial coaxial dipole implanted in a rabbit brain tangentially to the inner skull surface *in vivo*. The coaxial dipole had a contact at the tip that consisted of a 0.5 mm segment of exposed platinum wire of 0.25 mm diameter. The second ring shaped contact consisted of a 0.5 mm segment of the exposed platinum tube of 0.7 mm outer diameter. The distance between the contacts on the center axis was 1 mm, which resulted in a distance of approximately 1.1 mm between the contacts due to the conical shape. The dipole was connected to a constant-current source (20 Hz, 0.1 mA) with a sinusoidal waveform. Measured signals were band-pass filtered (15–25 Hz) and approximately 300 consecutive trials (sinusoidal waves) were averaged. The resulting signal-to-noise ratio was above 40 dB, meaning that noise can be considered negligible in the analysis.

Following a reference recording with intact skull, two skull defects were introduced above the dipole and filled with conducting agar (1.0 S/m at 30°C). The dipole was shifted inside the cortex under one of the skull defects (defect 1) in regular steps and measurements were taken at each step. The shifting was achieved by rotating a screw on the fixation device. One full rotation resulted in approximately 0.7 mm shift, called “full step,” and half of a rotation in a 0.35 mm shift, called “half step.” We numbered the shift positions in 0.35 mm steps. Because the *in vivo* experimental setup had limited stability over time, we measured at full steps, i.e., every second shift position, except under one of the edges of defect 1 where the field-maps changed rapidly. To avoid tissue damage and bleeding that would compromise the results, the dipole implant was only moved in once to the skull defect site while the skull was intact. Then, after introducing the skull defects, the dipole implant was shifted to the end of its range and stepwise back out under the defects. Consequently, intact skull recordings only exist for one side of defect 1, e.g., in **Figures 2A**, **5**, **6**. Further details of the experiment and measured signals are described in Lau et al. (2014).

The position of the artificial dipole was determined from post-experimental computed tomography (CT) with 0.4 mm isotropic voxels. The geometry of the skull defects was manually segmented from the CT. For each defect the outer edge, the inner edge and the middle of the cut surface was sampled finely to accurately represent its shape. We defined the shift eccentricity of a point on the shift line as 0 at the normal projection of the defect center on the shift line and +1 or −1 at the normal projections of the outermost edge points onto the shift line (see **Figures 3**, **5**).

Before the experiment, a T2-weighted anatomical MRI data set (Siemens Magnetom 3T (Siemens, Erlangen, Germany), 224 slices, slice thickness 0.4 mm, 256 × 194 in-plane with resolution 0.423 mm, TR 2500 ms, TE 337 ms) was acquired of the anesthetized rabbit using a NORAS 8 Channel Multifunctional Coil (CPC) (NORAS MRI Products GmbH, Höchberg, Germany) that consisted of four coils directly above the rabbit's head and four coils below it. The signal intensity drop off with distance to the coils was corrected using nonparametric non-uniform intensity normalization (N3; Sled et al., 1998).

The co-registration was optimized to approximately 0.1–0.2 mm using a custom stereotactic frame, in which the rabbit head was fixated. Four spherical CT-visible markers were attached to the skull as landmarks to co-register the CT and head model. A well-defined configuration of coils, which was permanently mounted to the base of the stereotactic frame, was used to co-register the MEG sensors. The position of the rabbit head was continuously monitored.

### Segmentation and meshing

The tissues of the head were segmented semi-automatically using Seg3D^1^ from the co-registered CT and MRI volume data sets. For computational efficiency, the ears, the neck, and the lower part of the head, consisting primarily of the jaw, were removed from the body compartment. The skull was segmented by thresholding the CT and manually correcting for the artifact of the dipole implant and discontinuities due to the limited voxel resolution. Natural skull openings toward the spine and at the optic nerve exit were modeled open. The burr hole at the posterior lateral region of the skull, through which the coaxial dipole was inserted, was closed in the segmentation, because it was sealed with plastic and non-conducting glue during the experiment. It was ensured that the cancellous bone was enclosed by compact bone.

The white matter, CSF, and major blood vessels were segmented using thresholding of the T2 volume data set and manual completion using an anatomical atlas of the rabbit brain. The CSF layer around the brain was much thinner than one voxel side length in the pre-experimental MR image and, during the experiment, the brain was pressing against the intact, almost transparent dura at the interior boundary of the skull defect. Consequently, no CSF layer around the brain was differentiated.

The skull defect geometries were manually segmented from the CTs as accurately as the voxel resolution allowed. The liquid layer was derived by dilating the skull by 1 voxel and subtracting the body (including the skull). The stem of the coaxial implant was not differentiated. The miniaturized coaxial design eliminated most interference.

**Figure 1:**
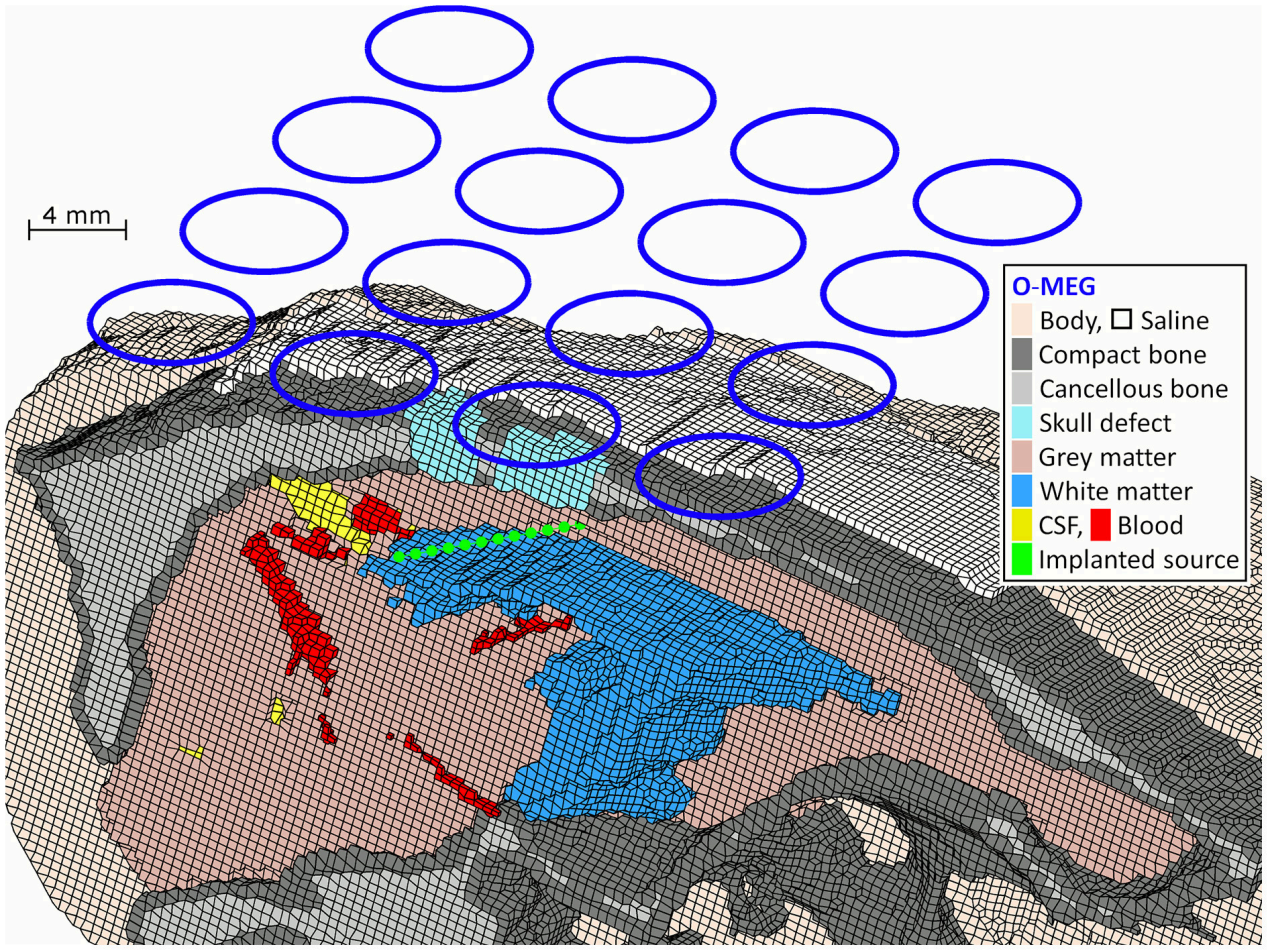
**FE model of rabbit's head showing MEG coils, experimental skull defects (defect 1 is the right one and defect 2 is the left one), source positions and tissue compartments that are cut open at different levels to expose internal anatomical structures**.

The segmentation was converted into a hexahedral mesh from the voxel coordinates using the software Vgrid version 1.3.1^2^ as shown in Figure 1. Using Vgrid, node-shifting (Camacho et al., 1997; Wolters et al., 2007) with a smoothing factor of 0.49 was applied to smooth the surfaces between compartments.

### Conductivities

Equivalent homogeneous conductivity values, summarized in Table 1, were assigned to each segmented tissue type as well as the agar in the skull defects and a layer of conducting liquid on the skull surface mimicking a thin layer of skin. These conductivity values were used for both forward simulations and source reconstructions. The tissue conductivity values were taken from the literature (Geddes and Baker, 1967). The scalp/body conductivity was chosen as a mixture of fat, skin, and muscle, weighting fat strongest due to the subcutaneous fat layer (Gabriel et al., 2009). For the inner and outer compact bone layers of the skull a conductivity of 0.004 S/m (Tang et al., 2008) was used. Because the cancellous bone consists of bone, fat, cells, and blood, an approximate value of 0.046 S/m (Haueisen et al., 1997; Akhtari et al., 2002, 2006) was used. Gray matter conductivity of 0.23 S/m was used (Crile et al., 1922; Akhtari et al., 2006). For white matter, an equivalent isotropic conductivity of 0.31 S/m was chosen, which lies between the lower conductivity perpendicular to the fibers (0.125 S/m measured by Nicholson, 1965) and the higher conductivity parallel to the fibers (1.18 S/m measured by Nicholson, 1965). The conductivity of the vitreous humor of the eye was measured to be 1.55 S/m at 25°C (Pauly and Schwan, 1964). Since the eyes of a rabbit are positioned mostly outside the skull, a temperature of 30°C was assumed and a conductivity of 1.55 S/m + 5^*^ 0.031 S/m = 1.7 S/m was used for the vitreous humor and the aqueous humor. The conductivity of the lens was estimated with 0.35 S/m based on the measurements of Pauly and Schwan (1964) and Lindenblatt and Silny (2001).

**Table 1:** **Tissue and material conductivities**.

**Tissue**	**Conductivity in S/m**
Scalp/body tissue	0.33
Compact bone	0.004
Cancellous bone	0.046
Gray matter	0.23
White matter (isotropic equivalent)	0.31
Cerebrospinal fluid	1.79
Intracranial blood vessels	0.78
Ocular humor	1.7
Ocular lens	0.35
Agar in defects	1.0
Liquid layer	0.6

### MEG coil geometries and positioning

The geometric setup of the *in vivo* rabbit experiment (Lau et al., 2014) was reproduced in the simulation setup as far as possible. The exact asymmetric MicroSQUID gradiometer array geometry (Nowak et al., 1999) was used, except for the base length of the gradiometers that had to be modeled uniformly with 30 mm instead of 28.5–31.5 mm to meet the requirements of the simulation software. The field-map magnitude and topography deviation between simulated non-uniform and uniform base length geometries in the experimental setup was small (*MAG*_rel_ < 0.001, RDM^*^ < 0.01).

### Forward and inverse solution methods

Various approaches have been developed to solve the MEG forward problem and model the source singularity. Based on recent comparison studies with regard to numerical and modeling accuracy as well as computational speed (Wolters et al., 2007; Lew et al., 2009; Vorwerk et al., 2014), we used the Venant direct FEM approach for modeling the dipole source (Buchner et al., 1997; Wolters et al., 2007) and trilinear basis functions in an isoparametric FEM approach in the geometry-adapted hexahedral volume conductor model (Wolters et al., 2007). This approach has a high computational efficiency when used in combination with the FE transfer matrix approach (Wolters et al., 2004) and an algebraic multigrid preconditioned conjugate gradient solver (Lew et al., 2009). We performed our computations using the SimBio software toolbox.^3^ In forward simulations, source strength of 130 μAmm was used, given that a constant current of 100 μA was injected, that the shortest distance between the electrodes was approximately 1.1 mm, and that the current spreads across the dipole contact surfaces to some degree.

Given the a priori knowledge of a single dipolar source, an unconstrained moving dipole fit was chosen to reconstruct the source properties from the *in-vivo* MEG measurements. To speed up convergence, the source position derived from the CT was used as the initial guess. Downhill simplex optimization (Nelder and Mead, 1965) was used (reflection factor −1.0, expansion factor 2.0, contraction factor 0.5, max. 200 iterations). The initial simplex size of 1 cm was chosen to match the dimensions of the rabbit brain, which is approximately 3 cm wide from left to right.

A limitation of the MicroSQUID gradiometer array was that it is mono-directional. This means that source components that are oriented normal to the MEG coil plane are not well detected (Haueisen et al., 2012). Consequently, after source reconstruction, the source orientation component that is normal to the MEG coil plane is not well determined by the measurement data of this system. The experiment was designed so that the implanted source was oriented almost parallel to the MEG coil plane (angle approximately 3°). Therefore, orientation components normal to the MEG coil plane were minimal and could be excluded from the analysis by setting this orientation component of the equivalent source to zero.

### Configurations for source reconstruction

During source reconstruction, three types of configurations were used (see **Figures 5, 6**):

1. Reference (*i-I)*: The intact skull measurements (denoted *i*) were reconstructed using the intact skull FE model (denoted *I*). This configuration is color-coded in green.
2. Ignoring skull defects (*d-I*): The measurements in the presence of skull defects (denoted *d*) were reconstructed using the intact skull FE model. This configuration is color-coded in red.
3. Incorporating skull defects (*d-D*): The measurements in the presence of skull defects were reconstructed using the FE model incorporating the skull defects. This configuration is color-coded in blue.

### Field-map measures

To quantify the topographical deviation caused by defect 1 and by the combination of both skull defects, we determined the relative difference measure RDM^*^ (Meijs et al., 1989)

(1) $$\begin{array}{r} RDM^{*}=\sqrt{\overset{m}{\underset{i=1}{\sum }}{\left(\frac{v_{i,r}}{\sqrt{\overset{m}{\underset{j=1}{\sum }}v_{j,r}^{2}}}-\frac{v_{i,s}}{\overset{m}{\underset{j=1}{\sum }}v_{j,s}^{2}}\right)}^{2}}, \end{array}$$

where *i* and *j* are the channel indices, *v*_*i, r*_ is the value of the reference signal with intact skull, and *v*_*i, s*_ is the value of the signal measured with either one or two defects. The RDM^*^ is a goodness of fit measure that indicates the difference of two multichannel data vectors, such as two MEG field maps. Both sample vector values are divided by the respective vector norm to exclude scaling differences, which relate to the source strength rather than location and orientation. The RDM^*^ ranges from 0 to 2, where 0 means that the multichannel data vectors are identical and 2 means that one is the inversion of the other. To quantify the magnitude deviation caused by these two conditions, we determined the relative magnitude difference *MAG*_rel_ (Güllmar et al., 2006):

(2) $$\begin{array}{r} MAG_{rel}=\frac{\sqrt{\overset{m}{\underset{i=1}{\sum }}v_{i,s}^{2}}}{\sqrt{\overset{m}{\underset{i=1}{\sum }}v_{i,r}^{2}}}-1. \end{array}$$

For the comparison of simulated intact skull field maps with simulated skull defect field maps in the shift series, the reference map was the respective intact skull field map with the source at the same position as in the skull defect field map being evaluated. In the *in vivo* rabbit experiment, intact skull field maps could only be acquired for few of the shift positions. Therefore, the reference map for the comparison of measured field maps was the intact skull field map obtained with the dipole positioned close to the center of the subsequently introduced defect 1.

### Equivalent source characteristics

The reconstructed positions of the sources were characterized with the Euclidean distance to the physical position during the experiment. Taking into account the radius of the outer contact of 0.4 mm, the expected ideal value range for the Euclidean distance was assumed to be 0 to 0.4 mm. The spacing of equivalent sources along the shift line was characterized with the distance to the equivalent source one full step along the shift line. The expected ideal value is the physical distance of sources of 0.7 mm. The angle between the equivalent source orientation and the physical source orientation was evaluated. Angle variations of up to 16 degrees were within the expected ideal range, because the conic shape of the coaxial dipole surface had an angle of 16° between the center line and the line connecting the inner and outer platinum contact. The strengths of the equivalent sources were evaluated relative to an expected ideal range of 100–130 μAmm, given that a constant volume current of 100 μA was flowing between the platinum contacts that were approximately 1.1 mm apart at their closest points. The explained variance of the reconstruction was reported.

Two groups of physical source positions were selected for comparison (see, e.g., **Figure 5**): (1) source positions under defect 1 (shift eccentricity −0.5 to +0.5, shift position 15–22) were marked with a square, (2) source positions next to defect 1 and 2 (shift position 6–10) were marked with a sphere.

### Sensitivity analysis

To investigate the sensitivity of the source reconstruction to tissue conductivities, the conductivities of compact bone, cancellous bone, gray matter, and white matter were varied by ±10, ±20, and ±50% (Table 2).

**Table 2:** **Tissue conductivity variations in S/m for sensitivity analysis**.

**Tissue**	**−50%**	**−20%**	**−10%**	**+10%**	**+20%**	**+50%**
Compact bone	0.0020	0.0032	0.0036	0.0044	0.0048	0.0060
Cancellous bone	0.0230	0.0368	0.0414	0.0506	0.0552	0.0690
Gray matter	0.1150	0.1840	0.2070	0.2530	0.2760	0.3450
White matter	0.1550	0.2480	0.2790	0.3410	0.3720	0.4650

## Results

### Forward simulations vs. *in vivo* measurements

**Figure 2:**
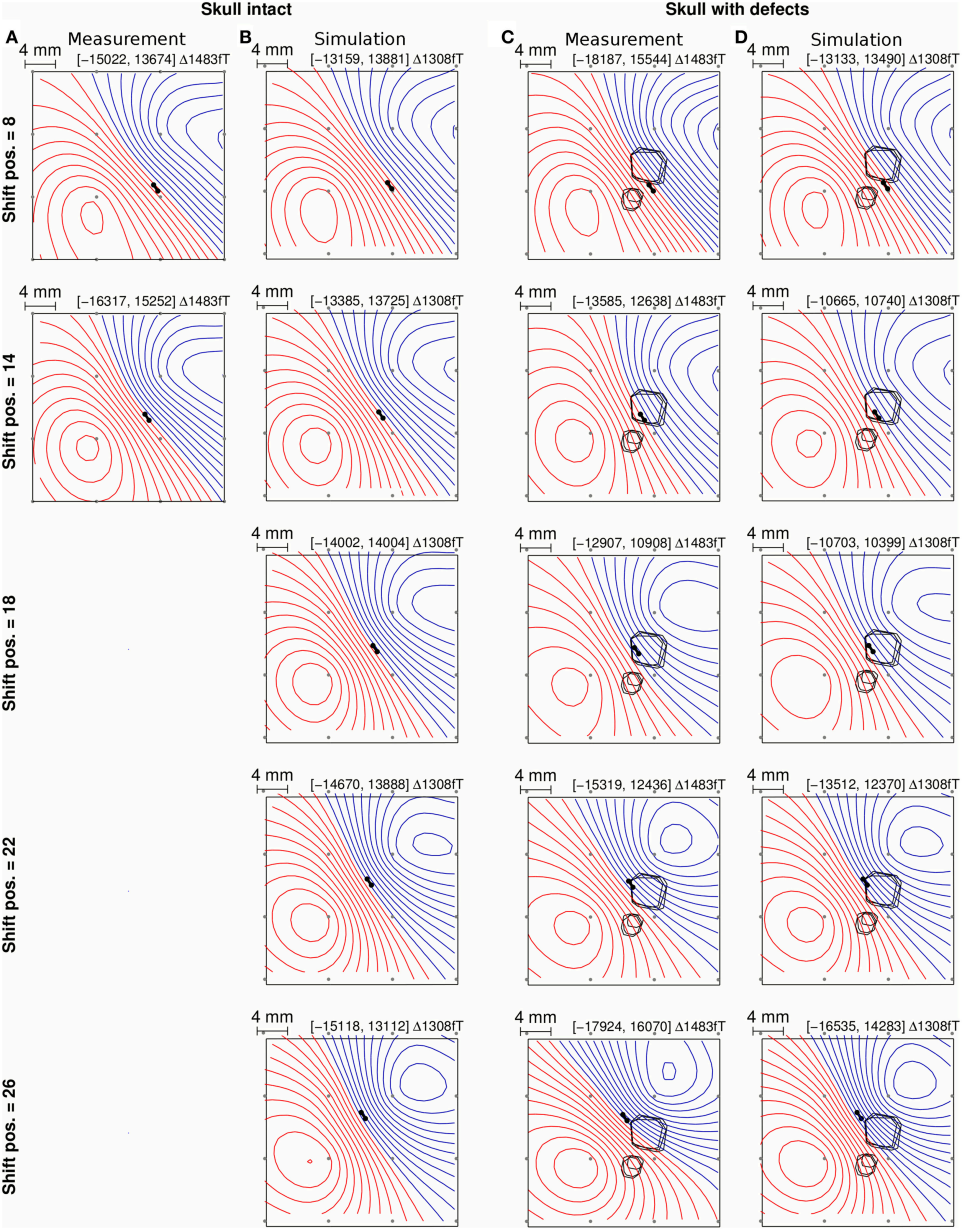
**Measured (A,C) and simulated (B,D) MEG flux density maps above intact skull (A,B) and above skull defects (C,D) for selected source positions relative to defect 1 (rows)**. The dipolar source is indicated with a black bar with two spheres marking the poles. Skull defects are marked by closed black lines indicating the inner, middle, and outer boundaries of the defects (see Figure 5 for a three-dimensional view). MEG coil positions are marked with gray dots. The minimum and maximum value (in fT) and the iso flux density line step are displayed above each map.

The MEG signals were simulated forward for a series of source positions along a linear path under defect 1, as shown in Figure 2D. These source positions matched the physical positions of the implanted artificial coaxial dipole during the *in vivo* measurements (Figure 2C). The simulations were repeated with the intact skull model (Figure 2B) to evaluate their concordance with the available intact skull recordings (Figure 2A).

The measured and simulated MEG signals above intact skull are shown side by side in Figures 2A,B for qualitative comparison. The gradual change in the measured flux density map topography as the source was shifted from one side of defect 1 to the other was reflected clearly in the simulated flux density maps (Figure 2C vs. Figure 2D). The absolute magnitude of the simulated flux density maps with constant source strength of 130 μAmm was within approximately ±30% of that of the recorded flux density maps (Figure 2).

**Figure 3:**
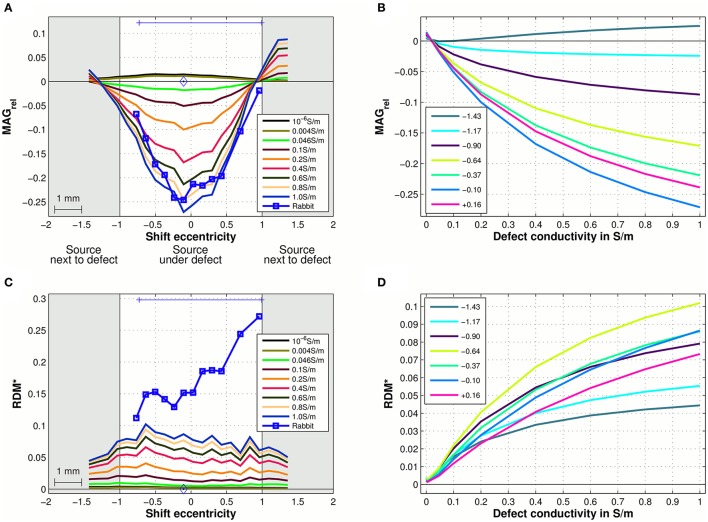
**MEG signal magnitude (A,B) and topography (C,D) differences between intact-skull condition and skull defect condition of the rabbit simulated for a range of defect conductivities. (A,C)** show the *MAG*_rel_ **(A)** and RDM^*^ **(C)** metrics on the shift eccentricity axis with the defect conductivity color-coded. Experimental data of the rabbit with physical skull defect conductivity 1.0 S/m is overlaid for comparison in **(A,C)** (blue squares). Due to limited availability of intact skull measurements, metrics of the measurements were calculated relative to one intact skull reference map (diamond marker) (see Lau et al. (2014)). The gray background indicates source positions next to the outermost extent of defect 1 on the shift line. The blue bar at the top of each diagram indicates the projected contact points of the shift line with the edge of defect 1 of the rabbit (see Figures 3, 5A in Lau et al. (2014)). **(B,D)** show the *MAG*_rel_ **(B)** and RDM^*^ **(D)** metrics with respect to defect conductivity for a selection of shift eccentricities (color-coded) from the center of defect 1 to the edge (indicated on the right of each curve).

Figures 3A,C shows the magnitude and topography differences between intact skull MEG signals and MEG signals above skull defects in measurement and simulation. In the measurements of the rabbit, the MEG signal magnitude dropped by approximately *MAG*_rel_ = 0.2 (20%) when the source was central under defect 1 (Figure 3A, blue square markers). The simulated MEG signals (Figure 3A, blue 1.0 S/m curve) showed the same magnitude reduction of approximately *MAG*_rel_ = 0.2 for the source central under defect 1.

The simulated topographic difference between the intact skull flux density maps and skull defect flux density maps (Figure 3C, 1.0 S/m) was approximately RDM^*^ = 0.07 for sources under defect 1. In the measurements of the rabbit, this difference was a bit larger (RDM^*^ approximately 0.16), which could be due to experimental variability. The slight discrepancy between measurement and simulation of RDM^*^ values for MEG at the lowest and highest shift positions is partly due to the fact that for the measured data one intact skull measurement (Figures 3A,C, diamond marker) close to the center of defect 1 was chosen as the reference from the limited set of available ones. In the simulation, intact skull MEG signals could be computed and used as the respective reference for all source positions. Therefore, the forward simulations reflect best the influence of skull defects on the magnitude and topography at the lowest and highest shift positions.

To evaluate the influence of the skull defect conductivity on the MEG signal change, the forward simulations were repeated with skull defect conductivities of 10^−6^ S/m (approximately air), 0.004 S/m (compact bone), 0.046 S/m (cancellous bone), 0.1 S/m, 0.2 S/m, 0.4 S/m, 0.6 S/m, and 0.8 S/m (Figure 3, color-coded). For skull defect conductivities below that of intact three-layer skull, i.e., 10^−6^ S/m and 0.004 S/m, the MEG signal magnitude was marginally increased (*MAG*_rel_ = 0.02) for a source under a skull defect. For defect conductivities above that of intact three-layer skull, the MEG signal magnitude was decreased. In Figures 3B,D, the simulated magnitude and topography differences due to the presence of the skull defects are plotted over the range of defect conductivity values. The MEG signal magnitude differences increased as the defect conductivity was increased and this effect was greater for small increases of the defect conductivity above that of skull (Figure 3B). The MEG signal magnitude change in this setup reached 50% of the largest observed magnitude change per source position in a range of approximately 0.2–0.3 S/m defect conductivity, which emphasized this non-uniformity. The flux density map topography change showed a similar non-uniform dependency and reached 50% of the largest observed topography change per source position also in a range of approximately 0.2–0.3 S/m (Figure 3D).

**Figure 4:**
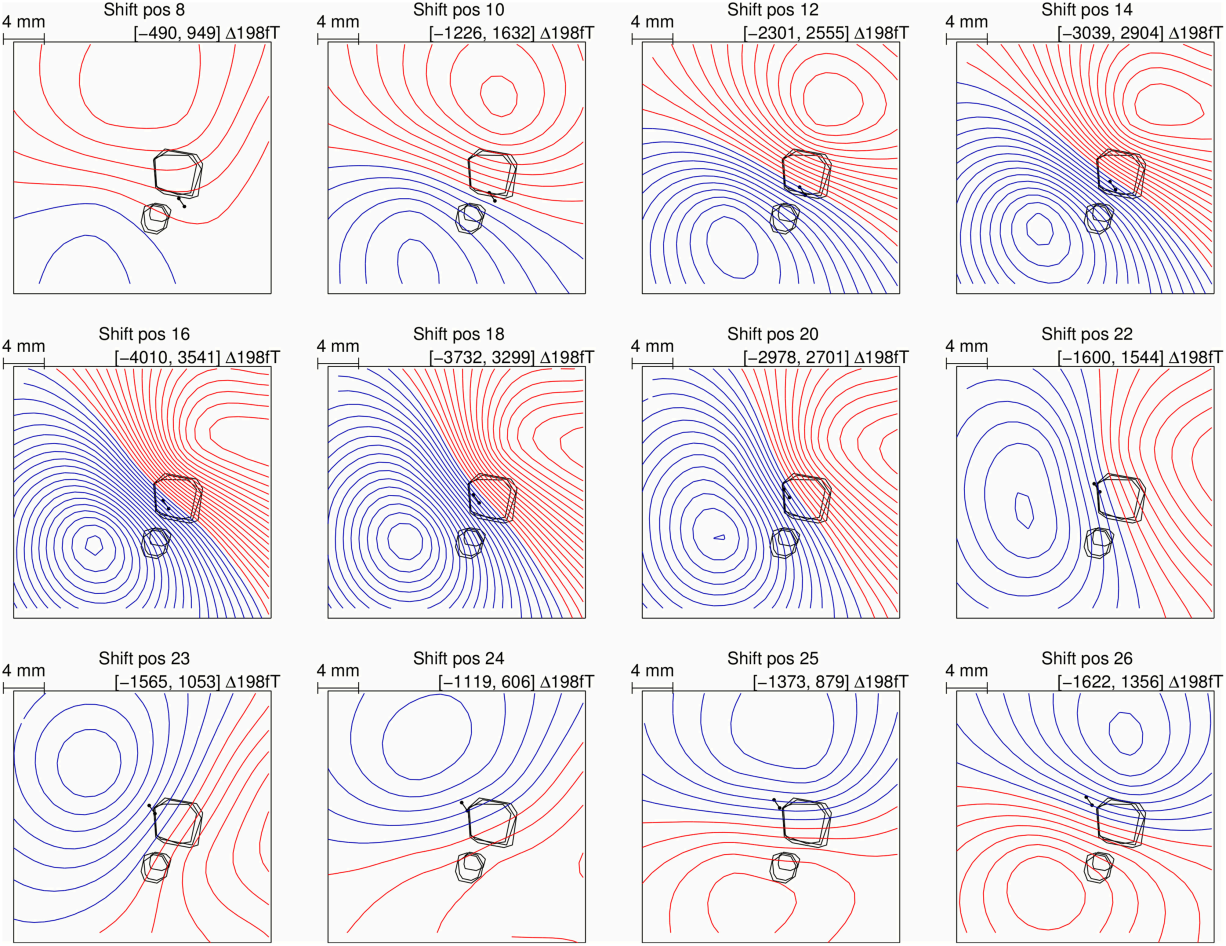
**Simulated flux density map differences between intact skull condition and skull defect condition for a range of source positions under defect 1 showing a gradual MEG topography change equivalent to the experimental observation (Figure 8 in Lau et al. (2014))**.

To investigate the nature of the topography change in the forward simulations, the intact skull flux density maps were subtracted from the skull defect flux density maps at different source positions, which are shown in Figure 4. The signal component caused by the skull defects was mainly dipolar and of approximately opposing orientation to the intact skull flux density map when the source was central under defect 1 (shift position 18). As the source was shifted from shift position 18–26, the orientation of the MEG signal component rotated. The same observation was made in the animal experiment (Figure 8 in Lau et al. (2014)).

### Source reconstruction from *in vivo* measurements

#### Intact skull FE model vs. skull defect FE model

**Figure 5:**
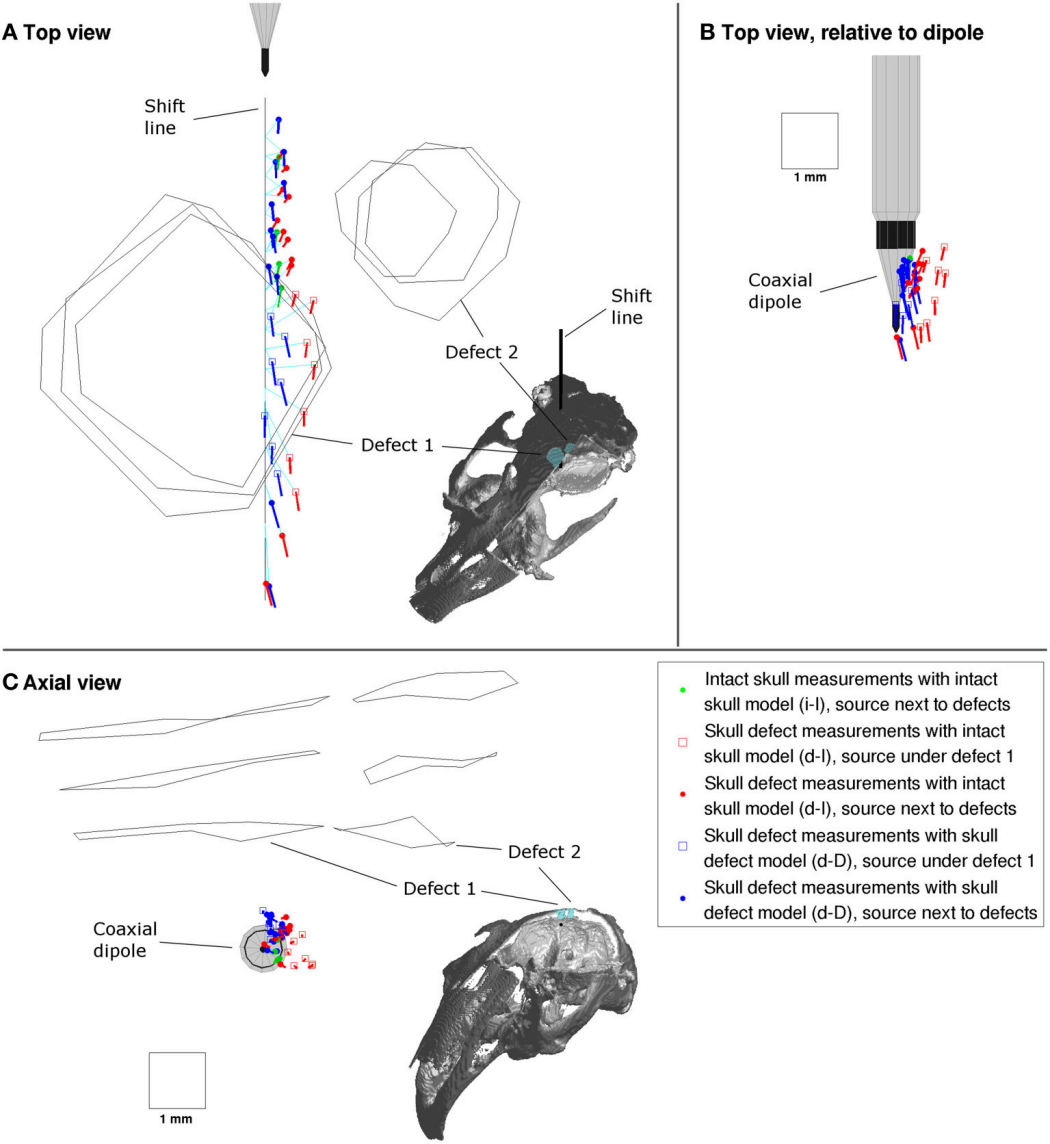
**Top (A,B) and axial view (C) of sources reconstructed from the successive MEG recordings along the shift line (black line)**. The inset images show the respective spatial view direction using the rabbit skull compartments, which are cut open to expose the inside of the skull. The dipole orientation line length is proportional to the source strength (1 mm = 300 μAmm). Sources reconstructed in *i-I* configuration in green, *d-I* configuration in red and *d-D* configuration in blue. Turquoise lines indicate mapping of equivalent to physical source position.

Figure 5 shows the equivalent single dipole sources reconstructed from the measured MEG signals. More than 99% (median 99.91% [lower quartile = 99.82%, upper quartile = 99.94%]) of the signal variance was explained by the forward simulation of the equivalent source in all reconstructions.

##### Intact skull measurements with intact skull model (i-I)

The intact skull equivalent dipoles (Figure 5, green) were arranged along a line, in the sequence of the shift positions and equidistant. The full step distances of equivalent sources were approximately 0.7 mm, which matched the physical distance of 0.691 mm. Their positions were offset from the physical positions by approximately 0.46 mm [0.44 mm, 0.48 mm]. The orientations of the intact skull equivalent dipoles pointed more toward the shift line with an angle to the shift direction around 6.3° [5.8°, 6.7°] (top view in Figure 5A). The equivalent source strengths were approximately 78 μAmm [69 μAmm, 87 μAmm].

##### Skull defect measurements with intact skull model (d-I)

The equivalent dipoles of the skull defect MEG measurements reconstructed using the intact skull FE model (Figure 5, red) were arranged in sequence along the shift line but not equidistant. The sources next to the defects (round markers) had an offset from the shift line of approximately 0.51 mm [0.43 mm, 0.53 mm], which is comparable to the *i-I* result. Sources under defect 1 (square markers), however, were displaced radially away from defect 1, partly to the side of defect 1 (Figures 5A,C) and partly deeper (Figure 5C). The offsets from physical positions were around 0.92 mm [0.80 mm, 1.02 mm]. The orientations of the equivalent sources next to the defects were substantially altered to angles of 42° [35°, 44°] relative to the shift direction. The sources under defect 1 were oriented similarly to the *i-I* result with angles of 8.1° [3.4°, 12.4°]. The equivalent dipole strength of sources next to the defects was reduced to 41 μAmm [37 μAmm, 45 μAmm], while under defect 1 the strength was comparable to the *i-I* results with 80 μAmm [75 μAmm, 86 μAmm].

##### Skull defect measurements with skull defect model (d-D)

When the exact FE head model incorporating the skull defects was used to reconstruct the skull defect MEG signals (Figure 5, blue), the equivalent dipoles of were arranged in a line passing under defect 1. The distances to physical positions under defect 1 of 0.58 mm [0.48 mm, 0.71 mm] were close to those of sources next to the defects of 0.39 mm [0.36 mm, 0.45 mm]. The full step distances of equivalent sources of 0.75 mm [0.65 mm, 0.78 mm] next to defects and 0.91 mm [0.82 mm, 1.07 mm] under defect 1 indicate a small residual spatial dispersion of sources compared to the intact skull condition. The relative orientation of equivalent sources next to defects of 3.7° [1.2°, 5.7°] and under defect 1 of 8.4° [2.9°, 11.8°] was comparable to the *i-I* result. The equivalent dipole strengths of sources next to the defects of 86 μAmm [79 μAmm, 88 μAmm] was comparable to the *i-I* result, while equivalent sources under defect 1 were a fraction stronger with 118 μAmm [110 μAmm, 129 μAmm].

To investigate the global offset of equivalent sources from the shift line, all equivalent sources were plotted relative to the physical dipole at the time of recording (Figure 5B). The *d-I* reconstructions of sources under defect 1 (red squares) were displaced from the implant surface, while the remaining sources were arranged close to the conic implant surface.

#### Defect conductivity variation

**Figure 6:**
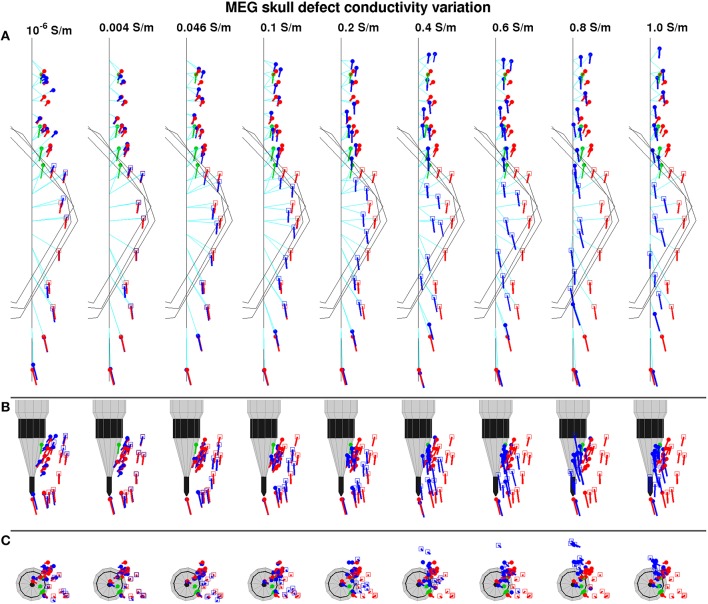
**Top view (A), relative to dipole view (B), and axial view (C) of equivalent sources reconstructed from measured flux density maps using a range of defect conductivities in the head model**. Markings are the same as in Figure 5.

Figure 6 shows the influence of the defect conductivity in the model on the equivalent sources. For defect conductivity 0.004 S/m, the equivalent sources reconstructed using the skull defect model (*d-D* reconstruction, blue) were positioned and oriented most similarly to the intact three-layer skull model (*d-I* reconstruction, red) (Figure 6). For defect conductivity 10^−6^ S/m (quasi-non-conducting), the displacement of equivalent sources was stronger (Figure 6, column 1). As the defect conductivity increased toward the experimental value of 1.0 S/m, the equivalent sources under defect 1 were positioned increasingly on a line (Figure 6). The distance to the physical positions (Figure 6) of equivalent sources diminished as the defect conductivity of the model approached that of the experiment, with the greatest change at the initial conductivity steps (0.4 S/m and below). The orientations of equivalent sources next to the defects approached the shift direction as the defect conductivity increased, with the greatest change for small increases of the defect conductivity. Equivalent sources under defect 1 were only marginally reoriented. The strength of the equivalent sources non-uniformly increased as the defect conductivity increased.

## Discussion

### Concordance of measurement and simulation

The forward simulation of the flux density maps reflects all topographic and magnitude features that were observed in the *in vivo* animal experiment. In particular, the gradually changing flux density map difference between the intact skull and the skull defect condition observed in the *in vivo* experiment (Figure 8 in Lau et al. (2014)) was also observed in the FE simulation (Figure 4). This agreement confirms that the observed changes pertain to the skull defects and are not due to experimental or modeling limitations. The conducting medium occupying the skull defect volumes represents a conducting bridge into and through the skull. This causes a displacement of the volume currents into and through the defect.

The magnitude difference between the measured and simulated flux density maps in the intact skull condition is likely due to experimental variability of the tissues around the implanted source. The intact skull recordings at the start of the experiment required approximately 15% higher voltage than the skull defect recordings several hours later to produce the same current. This means that the net resistance around the source was higher, i.e., the net conductivity was lower. However, the current remained constant.

In agreement with the *in vivo* experiment, the simulated flux density maps above conducting skull defects were approximately 20% weaker than the intact skull equivalent for sources that were central under defect 1. The magnitude reduction was diminished toward the boundaries of defect 1 in both measurement and simulation (Figure 3A). This confirms that a detailed FE model with conducting skull defects can quantitatively represent the influence of skull defects. A FE simulation of a neonate head (Lew et al., 2013) differentiating a single-layer non-ossified skull (conductivity 0.04 S/m) from fontanels and sutures (conductivity 0.3 S/m) also produced a change of MEG signal magnitude of up to absolute *MAG*_rel_ = 7.9% due to the presence of the fontanels and sutures. Considering that the neonate skull was very thin and had comparatively high homogeneous conductivity, and that the absolute *MAG*_rel_ was calculated over the whole head, including large field-map areas distant from defect 1, these absolute values are in accordance with our results.

The conductivity of the skull defects has a non-uniform effect on the flux density map change due to skull defects (Figure 3B). Small increases of the defect conductivity above that of intact three-layer skull have the strongest effect. The presence of a higher conducting path through the weakly conducting skull is the dominant reason for the signal change. This was described as the shunting effect (Brody, 1956; Rush and Driscoll, 1968; Haueisen et al., 1997). The MEG signal magnitude and topography change in this setup reached 50% of largest observed magnitude and topography change per source position at defect conductivities of 0.2–0.3 S/m, which is in the range of soft tissues.

Non-conducting skull defects, such as air-filled ones during surgery, have comparatively little influence on the flux density map. Our simulation of skull defects with conductivity 10^−6^ S/m caused a flux density map magnitude increase of less than 2%. Similarly to intact skull, the non-conducting defects represent a barrier to the volume current. When the defect conductivity approaches zero, the small amount of volume current that could have passed through the skull is also displaced into the brain volume. This is consistent with earlier observations involving non-conducting skull defects (Barth et al., 1986; Okada et al., 1999a,b).

An early simulation of human MEG using a spherical finite element head model (Van den Broek et al., 1998) investigated the flux density map topography above a skull defect of 3 cm diameter generated by tangential sources next to the skull defect at different depths and found no topography change. Their source positions are approximately equivalent to shift eccentricity ±1.3 (next to skull defect) in our study, where the influence of the skull defect is small in our study as well.

### Source reconstruction from measurements

#### Intact skull FE model vs. skull defect FE model

The reconstruction from the intact skull flux density maps (*i-I* configuration) serves as a reference condition. The full step distance of equivalent sources of 0.7 mm matches the physical distance of 0.69 mm very well. This indicates internal consistency of the model. The equivalent sources are arranged at the boundary of the conic surface of the implant tip (Figure 5B) with an effective distance to the shift line that is comparable to the outer radius of approximately 0.4 mm of the larger platinum contact. This indicates that the volume current was not completely symmetric around the shift line due to differences in the conductivity of the tissues surrounding the implant. The observed small angle between shift line and equivalent source orientations of approximately 6° can then be understood as a consequence of the conic shape of the physical source (angle of 16° to the shift direction).

Ignoring conducting skull defects in a head model (*d-I* configuration) can cause errors in position, orientation and strength of equivalent sources reconstructed from MEG signals. Sources under a skull defect are mostly displaced away from the defect; partly toward the boundary of the defect and partly deeper (Figures 5A,C). This matches the reduced MEG signal magnitude above the defect (Lau et al., 2014). When the skull defect is ignored, the source should be estimated farther away from the defect. In this case, the equivalent sources were displaced under or next to the boundary of the skull defect (Figure 5A). This displacement might scale to larger skull defects. The larger a skull defect, the more dominant is its influence on the flux density map and consequently on the equivalent sources. Sources next to a skull defect are mostly reoriented; in this experiment by approximately 35°. This is explained by the reduced MEG signal magnitude above the defect causing a net reorientation in the flux density map. Such orientation errors should be observable in sources that are proximal to skull defects in humans. The strength of equivalent sources next to the defects was underestimated by approximately half due to ignoring the skull defects.

Van den Broek et al. (1998), in their simulation study, used a spherical finite element head model with an intact skull layer to reconstruct sources next to a skull defect (equivalent to shift eccentricity ±1.3), which was present during forward simulation of the MEG signals. In agreement with our study, they found no location errors. The fact that they did not observe orientation errors is likely due to the complete symmetry in their model. The volume conductor was spherical, the defect had a circular shape, the source orientation was in plane with the center of the defect and the source was exactly tangential to the spherical skull surface.

A FE simulation study (Lew et al., 2013) of a neonate head with fontanels and sutures also produced position, orientation and magnitude errors in source reconstruction from MEG signals due to ignoring the fontanels and sutures. However, because of the much smaller conductivity difference between the thin non-ossified skull and the fontanels and sutures, their errors were smaller relative to the skull defect sizes.

Modeling skull defects in a realistic FE head model can compensate the errors in position, orientation and strength of equivalent sources. In our study, the displacement of equivalent sources away from defect 1 was corrected to reflect the linear sequential arrangement of physical sources. A small spatial dispersion of sources under defect 1 remained, which indicates the mesh resolution of the model may be at the limit that is necessary to exactly represent the fine defect compartment boundary. The mesh size was derived from highest available CT imaging and MRI resolution of 0.4 mm^3^. The benefit of a smaller element size derived from even higher resolution volume imaging should be investigated further. The orientation error of sources that are physically next to the defects was fully compensated by the incorporation of the skull defects in the model (Figure 5A, blue dots). The strength of these sources was restored from approximately half (41 μAmm) of the *i-I* reference strength (78 μAmm) to a value very close to it (80 μAmm).

#### Role of defect conductivity

The modeled defect conductivity non-uniformly modulates the influence of a skull defect on the position, orientation and strength of sources reconstructed from MEG signals. Small increases of defect conductivity above that of compact bone in the model most strongly reduce the position, orientation and strength error. This corresponds to the finding that the forward simulated flux density map changes most strongly for small increases of the defect conductivity above that of compact bone (Figure 3). Already for small defect conductivity values, e.g., below 0.2 S/m, the forward simulation explains most of the measured flux density map. In humans, non-acute skull defects would be occupied by tissue with conductivity around or below 0.2 S/m. Consequently, the demonstrated source reconstruction errors may be observed in humans. In contrast to the orientation and strength error, the position error of sources under a defect only gradually reduces as the defect conductivity in the model approaches the physical one (Figure 6). For accurate localization of sources under skull defects, the modeled defect conductivity should be chosen to match the physical defect conductivity.

#### Sensitivity to tissue conductivities

The variation of compact bone, cancellous bone, gray matter, and white matter tissue conductivities (not shown due to volume) confirmed that the values derived from measurement literature (Section Conductivities) are appropriate and that the source reconstruction results from MEG signals are stable in an environment of at least ±10% around the individual conductivity values. Larger changes of the conductivity of an individual tissue type gradually displaced the overall set of equivalent sources along the superior-inferior axis by small amounts. Gençer and Acar (2004), using their simulated voxel-wise maps of the sensitivity of the MEG signals to tissue conductivity changes, found that MEG signals, and with them their source reconstruction, are more sensitive to changes of the conductivity of the skull in the vicinity of the source. This is in accordance with our results.

The results indicate that strong changes to tissue conductivities of ±20% or more can influence source reconstruction from MEG signals and that tissue conductivities should be chosen as accurate as possible, which is in agreement with a sensitivity study of Van Uitert et al. (2004) using a human head shape. A small variation of the compact bone conductivity had comparatively little influence on source positions, orientations and strengths, which was also found by Stenroos et al. (2014). This further supports the observation that the origin of the source reconstruction errors is the presence of a highly conducting path through a weakly conducting compact bone layer, rather than the particular conductivity values. The influence of cancellous bone conductivity on position is stronger for a model with skull defect than a model without skull defects. This indicates that the skull defect allows the volume current to pass through the cancellous bone layer. Consequently, it is important to differentiate cancellous and compact bone layers in head models that involve post-surgical skull defects.

### Modeling skull defects in humans

The source reconstruction errors due to ignoring skull defects in adult human MEG investigations are expected to be substantial. Human post-surgical skull defects are usually larger than in this experiment, in absolute size as well as relative to the skull surface. Increasing the skull defect size is known to cause a more substantial displacement of volume currents, a wider spread of electroencephalography (EEG) signal changes (Van Burik and Peters, 2000; Oostenveld and Oostendorp, 2002) and larger EEG signal source reconstruction errors (Lanfer et al., 2012). The same principle applies to MEG signals. The increased displacement of volume currents causes wider spread MEG signal changes, which result in larger source reconstruction errors. The neonate human skull should be differentiated from the adult one, because it is much thinner and of higher conductivity. The fontanels and open sutures present a weaker conductivity contrast to the non-ossified skull and consequently their influence should be weaker than in adults. This is supported by an EEG source reconstruction study in five neonates (Roche-Labarbe et al., 2008) as well as a MEG-EEG finite element simulation using a neonate head (Lew et al., 2013).

Sources close to a skull defect need to be differentiated from distant sources. Ignoring skull defects during source reconstruction from MEG signals displaces sources that are physically under a defect away from that defect. Sources physically next to a skull defect are reoriented and reduced in source strength. While in humans, the types of source reconstruction errors are the same, the much larger skull defect volume and large head surface portion of a skull defect is expected to cause larger position, orientation and strength errors than in this study. These errors occur at defect conductivities in the range of human soft tissue. Consequently, natural skull openings, such as the orbital fissures, should also be included in head models. The conductivity of post-surgical skull defect tissue over time should be investigated further, because it has a non-uniform influence on MEG signals. Further, the influence of model errors and skull defects, respectively, on the confidence region around the optimal source location and orientation should be investigated in human head geometry with whole-head sensor coverage.

The direct comparison of MEG measurements of a controlled source at known locations under a skull defect with an exact finite element forward simulation of the same setup validates the finite element approach to volume conductor head modeling in the presence of skull defects qualitatively and quantitatively. An alternative approach to head modeling is the boundary element (BE) method, which is based on a discretization of tissue compartment boundaries. The standard BE approach had been to assume nested compartments, such as scalp, skull, and brain (Kybic et al., 2006). This partly allowed for modeling skull defects in EEG (Bénar and Gotman, 2002; Oostenveld and Oostendorp, 2002) either by joining the scalp, defect, and brain compartment (Van Burik and Peters, 2000), by thinning the skull compartment at a skull defect (Roche-Labarbe et al., 2008), or by introducing an extra defect compartment inside the skull layer. Recent developments of the BE method relaxed the limitation of nested compartments for EEG (Kybic et al., 2006; Stenroos and Sarvas, 2012) and MEG (Stenroos and Sarvas, 2012). The application of BE approaches to skull defects in MEG should be investigated further and validated.

The spatial accuracy of source reconstruction from human MEG signals is determined by a combination of factors, including signal to noise ratio, spatial sampling density, co-registration accuracy, sensor coverage, and head model simplifications such as ignoring skull defects. In our controlled experiment, we isolated the influence of skull defects by minimizing the impact of all other factors. We showed that realistically incorporating skull defects in head models enables us to reconstruct sources from MEG signals recorded in the presence of skull defects. In a human application setting, this approach reduces the compound error by eliminating one error source. MEG studies should account for conducting skull defects during pick-up coil placement and modeling in the following way:

1. The MEG signal should be sampled densely in the proximity of a skull defect to capture the higher spatial frequencies.
2. The co-registration accuracy of MEG coil positions relative to the head should be maximized.
3. The skull defect boundary should be segmented as exactly as possible, if available from a CT, because CT is not geometrically distorted and has a good bone-tissue contrast.
4. The types of tissue occupying the defect volume should be determined and differentiated carefully and matching conductivity values should be used.
5. The compact and cancellous bone layers adjacent to skull defects should be differentiated and modeled as exactly as possible, because of the leakage of current into the cancellous bone.

## Conclusion

Our study provides qualitative and quantitative evidence based on experimental measurements that ignoring skull defects in volume conductor head models can influence source reconstruction from MEG in terms of location, orientation, and strength of the equivalent sources. The skull defect conductivity has a non-uniformly modulating influence on the changes to signal and equivalent source. A detailed realistic finite element model incorporating skull defects can reproduce the influence of skull defects on the MEG signals. Realistic FE head modeling combined with appropriate instrumentation enables source reconstruction from MEG in the presence of skull defects.

We conclude that skull defects should be incorporated in volume conductor models of the head for source reconstruction from MEG signals. This is particularly important in post-surgical and post-traumatic cases in which the brain area under investigation is typically under or proximal to the skull defect. The conductivity of skull defect tissues should be investigated further. Our next step is to investigate the utility of exact FE head models in source reconstruction from EEG in the presence of skull defects using the same controlled-source experiment. This modeling approach can improve the diagnostic localization and characterization of brain activity, such as epileptic discharges, in post-surgical patients.

## Author contributions

SL and JH conceived the study. SL designed and developed the experimental setup and experimental methods. SL performed the data acquisition. DG facilitated the MR imaging of the animals. LF facilitated the anesthesia and surgical work on animals. SL processed the measured data and volume data sets. SL constructed the FE model and performed the simulations and source reconstructions. SL performed the analysis, produced the images, interpreted the results, and wrote the paper. All authors took part in the scientific discussion at multiple stages of the study and provided feedback from the experimental (JH, LF), modeling (JH, CW, DG), theoretical (JH, CW, DBG), and clinical (MC, DBG) perspective. All authors reviewed the manuscript and approved it for publication.

### Conflict of interest statement

The authors declare that the research was conducted in the absence of any commercial or financial relationships that could be construed as a potential conflict of interest.

## Footnotes

^1^Center for Integrative Biomedical Computing. (2015). *Seg3D: Volumetric Image Segmentation and Visualization*. Scientific Computing and Imaging Institute (SCI). Available online at: http://www.seg3d.org. [Accessed 1st August 2015]

^2^Berti, G. (2016). The SimBio-Vgrid Mesh Generator. Available online at: http://vgrid.simbio.de/. [Accessed 30th March 2016].

^3^SimBio Group (2015). *SimBio: A Generic Environment for Bio-Numerical Simulations*. Available online at: https://www.mrt.uni-jena.de/simbio. [Accessed 1st August 2015].

## References

[B1] Akhtari M. Bryant H. C. Mamelak A. N. Flynn E. R. Heller L. Shih J. J.. (2002). Conductivities of three-layer live human skull. Brain Topogr. 14, 151–167. 10.1023/A:1014590923185 12002346

[B2] Akhtari M. Salamon N. Duncan R. Fried I. Mathern G. W. (2006). Electrical Conductivities of the freshly excised cerebral cortex in epilepsy surgery patients; Correlation with pathology, seizure duration, and diffusion tensor imaging. Brain Topogr. 18, 281–290. 10.1007/s10548-006-0006-x 16858632

[B3] Barth D. S. Broffman J. Sutherling W. Beatty J. (1986). Magnetic localization of a dipolar current source implanted in a sphere and a human cranium. Electroencephalogr. Clin. Neurophysiol. 63, 260–273. 10.1016/0013-4694(86)90094-5 2419084

[B4] Bénar C. G. Gotman J. (2002). Modelling of post-surgical brain and skull defects in the EEG inverse problem with the boundary element method. Clin. Neurophysiol. 113, 48–56. 10.1016/S1388-2457(01)00714-3 11801424

[B5] Brody D. A. (1956). A theoretical analysis of intracavitary blood mass influence on the heart-lead relationship. Circ. Res. 4, 731–738. 10.1161/01.RES.4.6.731 13365085

[B6] Buchner H. Knoll G. Fuchs M. Rienäcker A. Beckmann R. Wagner M.. (1997). Inverse localization of electric dipole current sources in finite element models of the human head. Electroencephalogr. Clin. Neurophysiol. 102, 267–278. 10.1016/S0013-4694(96)95698-9 9146486

[B7] Camacho D. Hopper R. Lin G. Myers B. (1997). An improved method for finite element mesh generation of geometrically complex structures with application to the skull base. J. Biomechanics 10, 1067—1070. 10.1016/S0021-9290(97)00073-0 9391875

[B8] Crile G. W. Hosmer H. R. Rowland A. F. (1922). The electrical conductivity of animal tissues under normal and pathological conditions. Am. J. Physiol. 60, 59–106.

[B9] Gabriel C. Peyman A. Grant E. H. (2009). Electrical conductivity of tissue at frequencies below 1 MHz. Phys. Med. Biol. 54, 4863–4878. 10.1088/0031-9155/54/16/002 19636081

[B10] Geddes L. A. Baker L. E. (1967). The specific resistance of biological material – A compendium of data for the biomedical engineer and physiologist. Med. Biol. Eng. Comput. 5, 271–293. 10.1007/BF02474537 6068939

[B11] Gençer N. G. Acar C. E. (2004). Sensitivity of EEG and MEG measurements to tissue conductivity. Phys. Med. Biol. 49, 701–717. 10.1088/0031-9155/49/5/004 15070197

[B12] Güllmar D. Haueisen J. Eiselt M. Gießler F. Flemming L. Anwander A.. (2006). Influence of anisotropic conductivity on EEG source reconstruction: investigations in a rabbit model. IEEE Trans. Biomed. Eng. 53, 1841–1850. 10.1109/TBME.2006.876641 16941840

[B13] Haueisen J. Fleissig K. Strohmeier D. Elsarnagawy T. Huonker R. Liehr M.. (2012). Reconstruction of quasi-radial dipolar activity using three-component magnetic field measurements. Clin. Neurophysiol. 123, 1581–1585. 10.1016/j.clinph.2011.12.020 22321298

[B14] Haueisen J. Ramon C. Eiselt M. Brauer H. Nowak H. (1997). Influence of tissue resistivities on neuromagnetic fields and electric potentials studied with a finite element model of the head. IEEE Trans. Biomed. Eng. 44, 727–735. 10.1109/10.605429 9254986

[B15] Kybic J. Clerc M. Faugeras O. Keriven R. Papadopoulo T. (2006). Generalized head models for MEG/EEG: boundary element method beyond nested volumes. Phys. Med. Biol. 51, 1333–1346. 10.1088/0031-9155/51/5/021 16481698

[B16] Lanfer B. Scherg M. Dannhauer M. Knösche T. R. Burger M. Wolters C. H. (2012). Influences of skull segmentation inaccuracies on EEG source analysis. Neuroimage 62, 418–431. 10.1016/j.neuroimage.2012.05.006 22584227

[B17] Lau S. Flemming L. Haueisen J. (2014). Magnetoencephalography signals are influenced by skull defects. Clin. Neurophys. 125, 1653–1662. 10.1016/j.clinph.2013.12.099 24418220

[B18] Lew S. Sliva D. D. Choe M. S. Grant P. E. Okada Y. Wolters C. H.. (2013). Effects of sutures and fontanels on MEG and EEG source analysis in a realistic infant head model. Neuroimage 76, 282–293. 10.1016/j.neuroimage.2013.03.017 23531680 PMC3760345

[B19] Lew S. Wolters C. H. Dierkes T. Röer C. MacLeod R. S. (2009). Accuracy and run-time comparison for different potential approaches and iterative solvers in finite element method based EEG source analysis. Appl. Numer. Math. 59, 1970–1988. 10.1016/j.apnum.2009.02.006 20161462 PMC2791331

[B20] Lindenblatt G. Silny J. (2001). A model of the electrical volume conductor in the region of the eye in the ELF range. Phys. Med. Biol. 46, 3051–3059. 10.1088/0031-9155/46/11/319 11720363

[B21] Meijs J. W. H. Weier O. W. Peters M. J. Van Oosterom A. (1989). On the numerical accuracy of the boundary element method. IEEE Trans. Biomed. Eng. 36, 1038–1049. 10.1109/10.40805 2793196

[B22] Nelder J. A. Mead R. (1965). A simplex method for function minimization. Comput. J. 7, 308–313. 10.1093/comjnl/7.4.308

[B23] Nicholson P. W. (1965). Specific impedance of cerebral white matter. Exper. Neurol. 13, 386–401. 10.1016/0014-4886(65)90126-3 5847284

[B24] Nowak H. Gießler F. Huonker R. Haueisen J. Röther J. Eiselt M. (1999). A 16-channel SQUID-device for biomagnetic investigations in small objects. Med. Eng. Phys. 21, 563–568. 10.1016/S1350-4533(99)00088-0 10672790

[B25] Okada Y. Lähteenmäki A. Xu C. (1999a). Comparison of MEG and EEG on the basis of somatic evoked responses elicited by stimulation of the snout in juvenile swine. Clin. Neurophysiol. 110, 214–219. 10.1016/S0013-4694(98)00111-4 10210611

[B26] Okada Y. C. Lahteenmäki A. Xu C. (1999b). Experimental analysis of distortion of magnetoencephalography signals by the skull. Clin. Neurophysiol. 110, 230–238. 10.1016/S0013-4694(98)00099-6 10210612

[B27] Oostenveld R. Oostendorp T. F. (2002). Validating the boundary element method for forward and inverse EEG computations in the presence of a hole in the skull. Hum. Brain Mapp. 17, 179–192. 10.1002/hbm.10061 12391571 PMC6872070

[B28] Pauly J. Schwan H. P. (1964). The dielectric properties of the bovine eye lens. IEEE Trans. Biomed. Eng. 11, 103–109. 10.1109/TBME.1964.4502313 14254229

[B29] Roche-Labarbe N. Aarabi A. Kungolo G. Gondry-Jouet C. Dümpelmann M. Grebe R.. (2008). High-resolution electroencephalography and source localization in neonates. Hum. Brain Mapp. 29, 167–176. 10.1002/hbm.20376 17390314 PMC6871239

[B30] Rush S. Driscoll D. A. (1968). Current distribution in the brain from surface electrodes. Anesth. Analg. 47, 715–723. 10.1213/00000539-196811000-00016 4972743

[B31] Sander T. H. Knösche T. R. Schloegl A. Kohl F. Wolters C. H. Haueisen J.. (2010). Recent advances in modeling and analysis of bioelectric and biomagnetic sources. Biomedizinische Technik 55, 65–76. 10.1515/bmt.2010.027 20367324

[B32] Sled J. G. Zijdenbos A. P. Evans A. C. (1998). A non-parametric method for automatic correction of intensity non-uniformity in MRI data. IEEE Trans. Med. Imag. 17, 87–97. 10.1109/42.668698 9617910

[B33] Stenroos M. Hunold A. Haueisen J. (2014). Comparison of three-shell and simplified volume conductor models in magnetoencephalography. Neuroimage 94, 337–348. 10.1016/j.neuroimage.2014.01.006 24434678

[B34] Stenroos M. Sarvas J. (2012). Bioelectromagnetic forward problem: isolated source approach revis(it)ed. Phys. Med. Biol. 57, 3517–3535. 10.1088/0031-9155/57/11/3517 22581305

[B35] Tang C. You F. Cheng G. Gao D. Fu F. Yang G.. (2008). Correlation between structure and resistivity variations of the live human skull. IEEE Trans. Biomed. Eng. 55, 2286–2292. 10.1109/TBME.2008.923919 18713698

[B36] Van Burik M. J. Peters M. J. (2000). EEG and implanted sources in the brain. Arch. Physiolog. Biochem. 107, 367–375. 10.1076/138134551999121070515FT367 10916164

[B37] Van den Broek S. Reinders F. Donderwinkel M. Peters M. (1998). Volume conduction effects in EEG and MEG. Electroencephalogr. Clin. Neurophysiol. 106, 522–534. 10.1016/S0013-4694(97)00147-8 9741752

[B38] Van Uitert R. Johnson C. Zhukov L. (2004). Influence of head tissue conductivity in forward and inverse magnetoencephalographic simulations using realistic head models. IEEE Trans. Biomed. Eng. 51, 2129–2137. 10.1109/TBME.2004.836490 15605860

[B39] Vorwerk J. Cho J.-H. Rampp S. Hamer H. Knösche T. R. Wolters C. H. (2014). A guideline for head volume conductor modeling in EEG and MEG. Neuroimage 100, 590–607. 10.1016/j.neuroimage.2014.06.040 24971512

[B40] Wolters C. H. Anwander A. Berti G. Hartmann U. (2007). Geometry-adapted hexahedral meshes improve accuracy of finite-element-method-based EEG source analysis. IEEE Trans. Biomed. Eng. 54, 1446–1453. 10.1109/TBME.2007.890736 17694865

[B41] Wolters C. H. Grasedyck L. Hackbusch W. (2004). Efficient computation of lead field bases and influence matrix for the FEM-based EEG and MEG inverse problem. Inverse Probl. 20, 1099–1116. 10.1088/0266-5611/20/4/007

